# Manipulation and *In Vitro* Maturation of *Xenopus laevis* Oocytes, Followed by Intracytoplasmic Sperm Injection, to Study Embryonic Development

**DOI:** 10.3791/52496

**Published:** 2015-02-09

**Authors:** Kei Miyamoto, David Simpson, John B. Gurdon

**Affiliations:** ^1^Wellcome Trust/Cancer Research UK Gurdon Institute, University of Cambridge; ^2^Department of Zoology, University of Cambridge

**Keywords:** Developmental Biology, Issue 96, *Xenopus* oocyte, oocyte maturation, Intracytoplasmic sperm injection, embryonic development, maternal factors, maternal depletion, micromanipulation, gene interference

## Abstract

Amphibian eggs have been widely used to study embryonic development. Early embryonic development is driven by maternally stored factors accumulated during oogenesis. In order to study roles of such maternal factors in early embryonic development, it is desirable to manipulate their functions from the very beginning of embryonic development. Conventional ways of gene interference are achieved by injection of antisense oligonucleotides (oligos) or mRNA into fertilized eggs, enabling under- or over-expression of specific proteins, respectively. However, these methods normally require more than several hours until protein expression is affected, and, hence, the interference of gene functions is not effective during early embryonic stages. Here, we introduce an experimental system in which expression levels of maternal proteins can be altered before fertilization. *Xenopus laevis* oocytes obtained from ovaries are defolliculated by incubating with enzymes. Antisense oligos or mRNAs are injected into defolliculated oocytes at the germinal vesicle (GV) stage. These oocytes are *in vitro* matured to eggs at the metaphase II (MII) stage, followed by intracytoplasmic sperm injection (ICSI). By this way, up to 10% of ICSI embryos can reach the swimming tadpole stage, thus allowing functional tests of specific gene knockdown or overexpression. This approach can be a useful way to study roles of maternally stored factors in early embryonic development.

**Figure:**
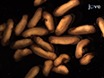


## Introduction

*Xenopus laevis* is a widely used, powerful model organism to study development^1^. This is because *Xenopus* eggs are unusually large (approximately 1.2-1.4 mm, in comparison to mammalian counterparts being about 0.1 mm) and abundant. Eggs contain maternally synthesized and stored components, which are enough to drive embryonic development until mid-blastula transition (MBT, occurring at the stage 8-8.5 with 4,000-8,000 cells). MBT is accompanied by zygotic genome activation, which then produces embryonic gene products that direct further development.

Numerous studies aimed to identify maternal factors important for development. Many studies rely on the injection of antisense oligonucleotides (oligos) including morpholino oligonucleotides into fertilized embryos, in which case degradation of maternal proteins can be observed at the gastrula stage^2-4^. Alternatively, mRNAs are injected to fertilized embryos to disturb gene functions or to trace fates of overexpressed proteins. However, the injection into the one-cell stage embryos normally does not affect expression levels of maternal proteins at the very early embryonic stages before MBT.

Heasman and Wylie established the host transfer method to overcome this issue^5^. In their method, manually defolliculated oocytes are injected with antisense oligos and transferred into host females^6^. Proteins are downregulated before fertilization so that roles of downregulated proteins in early embryonic development can be examined. This maternal depletion method led to the identification of several unique developmental roles of maternal proteins, as reviewed in ^7^.

In this report, we detail our recently developed method, in which maternal depletion or overexpression of mRNA before fertilization is achieved without manual defolliculation and host transfer^8^. Manual defolliculation requires a lot of time and host transfer often needs skillful techniques and the specific license for animal surgery, thus hampering the frequent use of maternal depletion method. Defolliculation and host transfer are substituted by enzymatic defolliculation ^9,10^ and intracytoplasmic sperm injection (ICSI)^11-13^, respectively. ICSI to eggs was originally used to produce transgenic frogs ^12^. Sperm and embryonic nuclei were also transplanted into *in vitro* matured amphibian oocytes ^11,14,15^. Here, we show our step-by-step method to inject sperm to *in vitro* matured oocytes that were pre-injected with antisense oligos or mRNA.

## Protocol

NOTE: All experimentation with frogs was carried out following requirements of the UK Home Office.

### 1. Preparation of *Xenopus laevis* Oocytes

1. Approximately a week before the oocyte collection, inject female frogs with 150 U of Pregnant Mare Serum Gonadotropin (PMSG) for pre-priming by using 1 ml sterile syringe with a 27 G needle. Injection is done subcutaneously into dorsal lymph sac. NOTE: It is optimal to use frogs that did not lay eggs for a long time (at least more than half year) or that have never laid eggs before.
2. Store pre-primed frogs in a water tank set at 18 °C in light controlled room (7 am–7 pm: on, 7 pm–7 am: off).
3. On the day of oocyte collection, prepare 1 L of 1x Modified Barth’s Solution (MBS) from 10x MBS stock by diluting with double distilled water (ddH_2_O),and add penicillin and streptomycin at the final concentration of 10 μg/ml each (MBS+P.S.). 10x MBS stock consists of 880 mM NaCl, 10 mM KCl, 24 mM NaHCO_3_, 8.2 mM MgSO_4_.7H_2_O, 3.3 mM Ca(NO_3_)_2_.4H_2_O, 4.1 mM CaCl_2_.6H_2_O, 100 mM HEPES, pH 7.5.
4. Anesthetize a pre-primed frog by subcutaneous injection of 120 mg (in 400 μl) of Tricaine methanesulfonate (MS222). Keep the injected frog in a small tank with water.
5. After 10 min, check the anesthetized frog by turning it over since successfully anesthetized frogs do not respond to this. If anesthesia is incomplete, add ice into the tank and wait for another 10 min.
6. Pick up the frog from the small tank and place in dorsal recumbency on a damp wipe.
7. Using forceps and a small surgical scissors, pinch the skin and make a small incision (2 cm) in the lower part of the abdomen, lateral to the midline. Make another incision at the other side.
8. After cutting the skin, lift the muscle layer with forceps and make the incision in the muscle layer.
9. Observe the ovary after the muscle layer is cut and pull the ovary out from the incisions using forceps.
10. Transfer extracted ovary to a 50 ml tube with MBS solution. NOTE: Try to collect as much ovary as possible from both incisions.
11. Slaughter the female frog by exsanguination, followed by freezing for subsequent appropriate disposal.
12. Rinse the extracted ovary a few times with MBS+P.S. until blood is washed away. Then, transfer the ovary to a 9 cm Petri dish containing MBS+P.S. NOTE: Check the quality of oocytes under a dissecting microscope at this point. If oocytes are apparently bad quality, such as oocytes with uneven pigmentation in the animal half (**Figure 1A**), do not process further and instead obtain a new ovary. Ideally, oocytes should have equally pigmented animal hemispheres and be approximately equally sized.
13. Tease the ovary into small pieces (1-2 cm^2^) and collect 5 ml of oocytes in a new 50 ml tube.
14. Rinse 5 ml of the torn ovary a few times with MBS+P.S. and add MBS+P.S. to 15 ml.
15. Add 7 units of the enzyme for defolliculation to the ovary suspension (see Materials List). NOTE: Reconstitute lyophilized enzyme with 10 ml of ddH_2_O (28 units/ml). Place the vial for 30 min on ice with occasional gentle swirling. Aliquot 250 μl and store at -80 °C until use.
16. Incubate the ovary piece with the enzyme for 1 hr with gentle shaking on the shaker (speed at 15 REV/min, equivalent to approximately 0.014 x g). NOTE: For almost complete removal of follicle cells, 2 hr to 2 hr 15 min incubation with the enzyme is normally needed. Longer incubation is needed for oocyte nuclear transfer experiment^16^.
17. After 1 hr incubation, pick up a small number of treated oocytes (10-20 oocytes) and transfer to a 6 cm Petri dish containing MBS+P.S. Check the extent of defolliculation under a dissecting microscope. If oocytes are separated from each other and at least partially defolliculated (**Figure 1B**), immediately proceed to the next stop reaction (step 18). If oocytes are still heavily surrounded by follicle cells, incubate for an extra 15 min at maximum.
18. Add plenty of MBS+P.S. to stop the reaction and discard the supernatant. Repeat the washing for 10 times. NOTE: Add MBS+P.S. by pouring to the wall of a 50 ml tube, but not directly to oocytes. After suspension in MBS+P.S., small immature oocytes tend to float at the top of washing buffer and discard such small floating oocytes.
19. After the washes, transfer to a 9 cm Petri dish containing MBS+P.S. NOTE: From this step onwards, keep oocytes at 16-18 °C as much as possible. This can be done by keeping the oocytes-containing dish on a temperature-controlled stage.
20. Select stage VI oocytes according to Dumont’s classification for subsequent uses and transfer to a new 9 cm Petri dish containing MBS+P.S. Oocyte transfer can be done by using a glass pipette with an appropriate tip size (bigger than the oocyte size). NOTE: Good quality oocytes should have an evenly pigmented animal hemisphere with clear contrast between the animal hemisphere and the vegetal hemisphere, and be approximately equally sized (**Figure 1C**). Stage VI oocytes represent fully grown oocytes that can respond to progesterone (oocyte diameter is about 1.2-1.4 mm).

### 2. Injection of Antisense Oligonucleotides or mRNA into Oocytes

NOTE: All steps in this section should be done at 16-18 °C on a microscope stage equipped with temperature-controlled circulating water or on a plastic box filled with ice.

1. Transfer 200-300 selected oocytes into a new 9 cm Petri dish filled with MBS+P.S., in which microinjection will be performed. NOTE: In each condition, 200-300 oocytes are normally used. In total, approximately 1,000 oocytes are used in one experiment.
2. For preparing injection of antisense oligos or mRNA, eject the metal plunger of a microinjector.
3. Fill a glass capillary with mineral oil using syringe and insert the glass capillary filled with oil to the metal plunger of microinjector. NOTE: A glass capillary is pulled by micropipette puller. These needles are kept in a box without damaging tips.
4. Cut the tip of the needle using small surgical scissors under a microscope by aiming as small diameter tip for injection as possible. NOTE: The needle tip can be sharpened by using a micro forge or micropipette beveler. To bevel the needle at an angle of 25° improves the capacity of penetrating the oocyte wall.
5. Place a small strip of Parafilm on the stage of a dissecting microscope and dispense a 3 μl drop of the antisense oligo or mRNA solution.
6. Move the tip of the injection needle into the small droplet of solution and fill the needle with the solution to be injected. NOTE: If the tip of the injection needle is too small, air bubbles should appear at the tip of the metal plunger. Then, make a bigger tip on the injection needle until this does not happen.
7. Mark the injection needle approximately 0.5 mm away from the tip. This mark is used as an indicator of injection depth.
8. Insert the tip of the needle into an oocyte along the equatorial boundary by aiming to the center point of the oocyte (underneath the germinal vesicle) while gently holding the opposite side of the oocyte to the injection point with forceps for preventing undesirable oocyte movement during injection. NOTE: Find the area free of follicle cells (**Figure 1B**) and insert the needle from that spot since even a fine needle cannot often penetrate a layer of follicle cells.
9. Eject 4.6 ng/4.6 nl or 9.2 ng/9.2 nl of antisense oligos, or an appropriate volume of mRNA (250 pg to 13.8 ng) by using the foot switch. Details of antisense oligo preparation are described in ^7^.
10. Transfer the injected oocytes into a 6 cm Petri dish filled with MBS+P.S. supplemented with 0.1% BSA (Oocyte incubation medium; sterilize the solution using a 0.45 μm pore filter).
11. Incubate the oocytes at 16°C or 18 °C for 1-2 days. NOTE: Injected oocytes can be subjected to *in vitro* maturation several hours after injection, in which case the injection of 4.6 ng antisense oligos is preferable. A general rule is that the shorter the incubation period is, the better the subsequent embryonic development.

### 3. *In Vitro* Maturation (IVM) of Oocytes

1. On the evening of the oocyte maturation experiment, fetch progesterone stock solution (30 mM in ethanol) from -80 °C freezer and dissolve precipitates at room temperature with occasional vortex. NOTE: The progesterone stock is normally left at room temperature for 30 min to 1 hr.
2. Add 5 μl of the progesterone stock in 50 ml of MBS+P.S. (Maturation medium; the final concentration of progesterone at 3 μM) and shake on the shaker for 30 min to distribute progesterone in the solution equally.
3. Prepare agarose-coated 6 cm dishes for *in vitro* maturation treatment.
  1. Add 1 g of agarose to 50 ml of 1x MBS without magnesium and calcium.
  2. Dissolve agarose by heating in a microwave.
  3. Pour a small volume of the agarose solution to 6 cm dishes to cover the bottom of dishes.
  4. Wait at least for 30 min until solidification.
  NOTE: Agarose coating prevents sticking of oocytes to dishes. Once *in vitro* matured oocytes are tightly attached to dishes, it is most likely that oocytes will be activated by the stimuli that they receive during the detachment from dishes.
4. Pour 5-8 ml of Maturation medium to agarose-coated dishes and transfer 200-300 oocytes into each 6 cm dishes. NOTE: Try to transfer oocytes with a minimal amount of oocyte incubation medium.
5. Incubate for 16 hr at 16 °C. NOTE: For example, start the treatment at 5 pm and finish at 9 am on the next day.

### 4. Intracytoplasmic Sperm Injection (ICSI)

1. Frozen sperm stock preparation NOTE: The following sperm preparation should be done before starting the oocyte maturation experiment, and frozen sperm stocks are kept at -80 °C for ICSI.
  1. Collect the testes from a male frog by following steps 1.4 to 1.11, except for pulling fat and testes out from the incisions using forceps and removing testes from the fat.
  2. Wash testes in 1x Marc’s Modified Ringers (MMR, 100 mM NaCl, 2 mM KCl, 1 mM MgSO_4_, 2 mM CaCl_2_, 5 mM HEPES, pH 7.4).
  3. Remove fat and blood vessels by rolling the washed testes on a clean tissue paper and then by removing remaining blood vessels using forceps.
  4. Place each testis in a 3.5 cm Petri dish containing 1 ml of 1x MMR.
  5. Cut longitudinally with fine scissors, and tear into small pieces with forceps until no big pieces remain.
  6. Pipette up and down using 1 ml plastic tip whose end is cut, and load 1 ml of the crushed testis solution into a glass homogenizer.
  7. Homogenize them by 2-3 strokes and then pour the solution onto a 50 μm pore filter attached to the top of a 15 ml centrifuge tube. Allow the solution to go through the filter.
  8. Repeat step 4.1.7 by resuspending the remaining cut testis in 1 ml of 1x MMR. Finally, wash the homogenizer a couple of times with 1x MMR and pass it through the filter.
  9. Repeat steps 4.1.5 to 4.1.8 for the other testis and pool two testes into one 15 ml centrifuge tube.
  10. Spin the cells at 800 x g at 4 °C for 20 min.
  11. Discard the supernatant and resuspend the pelleted cells in 2 ml of 1x MMR. Then, transfer into a new tube. NOTE: Make sure that no visible blood cells are present.
  12. Prepare a step gradient in a 14 ml ultra-clear centrifuge tube. Bottom layer: 4 ml of 30% iodixanol. Second bottom layer: 1 ml of 20% iodixanol. Third bottom layer: 5 ml of 12% iodixanol. Top layer: testis cells in 2 ml of 1x MMR. Overlay the gradients very gently with a 1 ml pipette tip (overlay slowly not to disturb the bottom phase). NOTE: Just one layer of 30% iodixanol should also work for isolating sperm only.
  13. Spin in the SW40 rotor using ultracentrifuge at 10,000 x g at 4 °C for 15 min (deceleration without a break).
  14. Gently remove the tube from the ultracentrifuge. Confirm an interface between each layer of the gradient and the pellet at the bottom.
  15. Collect the sperm fraction from the pellet.
  16. Resuspend the sperm pellet in 1x MMR for washing out iodixanol. Spin at 800 x g at 4 °C for 20 min. NOTE: If a lot of sperm still remain in the suspension after the spin, re-spin the supernatant at 3,220 x g at 4 °C for 20 min and pool the pelleted sperm.
  17. Discard the supernatant and resuspend the sperm pellet in 1 ml of 1x Nuclear Preparation Buffer (NPB; 250 mM Sucrose, 0.5 mM Spermidine trihydrochloride, 0.2 mM Spermine tetrahydrochloride, 1 mM EDTA, 15 mM HEPES, pH 7.7)^13^. Then, transfer to an Eppendorf tube.
  18. Add 50 μl of 10 mg/ml Digitonin solution in 1x NPB (Resuspend Digitonin powder in DMSO at 50 mg/ml and freeze aliquots at -80 °C. Before use, dilute the 50 mg/ml aliquot to 10 mg/ml with 1x NPB. Unused 10 mg/ml Digitonin can be frozen and stored at -20 °C and re-used) to 1 ml of the sperm solution.
  19. Incubate for 5 min at room temperature.
  20. Check a permeabilization rate by DAPI staining (0.3 µg/ml DAPI as a final concentration). If less than 95% of cells are permeabilized, incubate a bit longer. NOTE: Sometimes if too many sperm cells are incubated at the same time (especially when sperm are collected from multiple testes), it is difficult to permeabilize, in which case it may be necessary to add an additional small amount of Digitonin; for example if 20% of sperm are not permeabilized, an additional 20 μl of Digitonin is added.
  21. Stop permeabilization by adding 10% BSA to 3% final concentration.
  22. Spin the cells at 4 °C. NOTE: Try as little centrifugation speed as possible by starting with 100 x g for 5 min. If not sufficient, increase the speed and/or time.
  23. Wash the cells with 0.3% BSA in 1x NPB and centrifuge at the same speed as step 4.1.22. Meantime, prepare the Sperm Storage Buffer (SSB; 2 ml of 2x NPB, 120 μl of 10% BSA, 1.2 ml autoclaved Glycerol, 680 μl of H_2_O).
  24. Add 500 μl of SSB to the sperm pellet. Pipette several times up and down to resuspend with a cut tip in order not to damage the cells.
  25. Allow to equilibrate overnight at 4 °C.
  26. Pipette the cells up and down several times with a cut tip.
  27. Dilute a couple of microliters 10 times in 1x MMR or in 1x PBS to count the cells using hemocytometer.
  28. Freeze as aliquots at 30,000 sperm/μl (or higher) directly at -80 °C and store at -80 °C in a single use aliquots.
2. ICSI to *in vitro* matured oocytes NOTE: All processes involving oocytes should be done at 16-18 °C.
  1. Prepare a glass pipette for transferring matured oocytes. NOTE: The glass pipette has to be wide enough to accommodate oocytes without squeezing.
  2. Sixteen hours after moving oocytes to the progesterone-containing medium, transfer matured oocytes into a 6 cm agarose-coated dish filled with MBS+P.S. for washing. NOTE: Importantly, matured oocytes have to be treated extremely carefully. Rough treatment of oocytes may induce spontaneous activation before sperm injection.
  3. Count matured oocytes by confirming the appearance of white spots, which implies the germinal vesicle breakdown (**Figure 2**). If the maturation rate is less than 80%, it is unlikely to obtain the enough number of surviving embryos for further analyses. NOTE: Remaining follicle cells are peeled off after oocyte maturation (**Figure 2**).
  4. Transfer oocytes from MBS washing to a new agarose-coated 6 cm dish filled with injection medium (Prepare 500 ml: 30 g of Ficoll (6%), 20 ml of 10x MMR and 500 μl of 1,000x P.S. stock).
  5. Incubate at least for 30 min before starting ICSI.
  6. Set up the microinjector as described in steps 2.2-2.4. NOTE: The tip size of the injection needle is kept as small as possible by following the procedure described in step 2.6. The diameter of the needle is 20-40 μm. To bevel the needle tip as described in step 2.4 might allow efficient injection.
  7. Fetch the sperm aliquot and dilute the sperm suspension in Sperm Dilution Buffer (SDB; 250 mM Sucrose, 75 mM KCl, 0.5 mM Spermidine, 0.2 mM Spermine, 200 μM HEPES pH 7.5). NOTE: Dilution can be varied depending on a needle size and so on. Dilute 120 times of the 30,000 sperm/μl stock as an initial try and adjust further if needed.
  8. Place a small strip of Parafilm on the stage of a dissecting microscope. Mix sperm injection solution by pipetting and dispense a few μl drop of the sperm solution.
  9. Suck the diluted sperm suspension into the needle, inject 4.6 nl into 10 successive drops containing 0.3 µg/ml DAPI on a microscope slide, and quickly count the number of sperm delivered per injection on a fluorescent microscope. NOTE: Aim 1-2 sperm per injection. If necessary, dilute the sperm injection solution more and check the number of sperm per injection again until achieving a desired concentration.
  10. Eject the test injection solution and fill a new sperm injection solution with a proper concentration.
  11. Inject 4.6 nl of sperm solution to 100 matured oocytes. NOTE: It is important to continuously inject the solution. First determine the time required for ejecting 4.6 nl (normally 1-2 sec). Repeat the following process; inject in an oocyte, wait for a few seconds, remove a needle and mock-injection in injection solution, wait for a few seconds, inject in an oocyte. This continuous injection also prevents blocking of the needle tip. Alternatively, the method using a pump might be used^13^.
  12. After approximately 100 injections, eject the remaining sperm solution and re-fill the sperm solution (use the same sperm solution, but pipette up and down before dispensing). NOTE: If the needle does not suck sperm solution well, cut the tip of the needle. If this does not improve the situation, prepare a new needle.
  13. Repeat the injection process until all oocytes are injected. NOTE: If the oocyte quality is good and the injection is successful, contraction of injected oocytes is seen within 20 min of the injection time.
  14. Move the injected dishes to 16 °C or 18^ ^°C incubator.
  15. 4-5 hr after the injection, check the cleavage rate of ICSI embryos. NOTE: Cleavage furrows of those embryos may not be as clear as those of normal fertilized embryos. Some embryos also show abnormal cleavages, which might be caused by multiple sperm injection.
  16. Transfer cleaved embryos including abnormally cleaved embryos to incubation medium (Prepare 500 ml: 20 g of Ficoll (4%), 5 ml of 10x MMR and 500 μl of 1,000x P.S. stock).
  17. Incubate embryos either in 16^ ^°C or 18 °C incubator overnight.
  18. Next morning, transfer embryos to 0.1x MMR and count surviving embryos.
  19. On average, approximately 10% of sperm-injected oocytes can develop to the swimming tadpole stage (**Figure 3A**).

## Representative Results

Embryonic development of ICSI embryos using *in vitro* matured oocytes was examined (**Figure 3A**). Maturation rates of GV oocytes to the MII stage are variable and largely depends on the oocyte quality. In good experiments, almost 100% of GV oocytes respond to progesterone and show signs of oocyte maturation, finally becoming eggs at the MII stage. All matured oocytes were subjected to ICSI and about 25% of injected eggs cleaved (**Figure 3A**, n = 13 for control scrambled oligo-injected oocytes and n = 7 for no oligo-injected oocytes). Approximately 60% or 80% of cleaved embryos, produced from control oligo-injected oocytes or non-injected oocyte, respectively, reached the blastula/gastrula stage. Among the blastula/gastrula embryos, approximately half of embryos in oligo-injected samples were of good quality (almost no signs of abnormal cleavage and apoptosis) whereas 82% of blastula/gastrula embryos were of good quality in non-injected samples (**Figure 3A**). Finally, 41% and 11% of cleaved embryos in oligo-injected samples reached the muscular response and the swimming tadpole stages, respectively, while 60% and 29% of cleaved embryos in non-injected samples did (**Figure 3A**). These ICSI embryos are the mixture of normal and abnormal embryos (**Figure 3B**). Some of them undergo metamorphosis and develop to mature frogs^8^. These results suggest that injection of antisense oligonucleotides into GV oocytes, followed by IVM and ICSI, allows efficient early embryonic development. Although the injection of antisense oligos itself decreases embryonic development, we are still able to obtain enough embryos for many experimental purposes such as checking developmental rates, RT-PCR, western blot and so on.


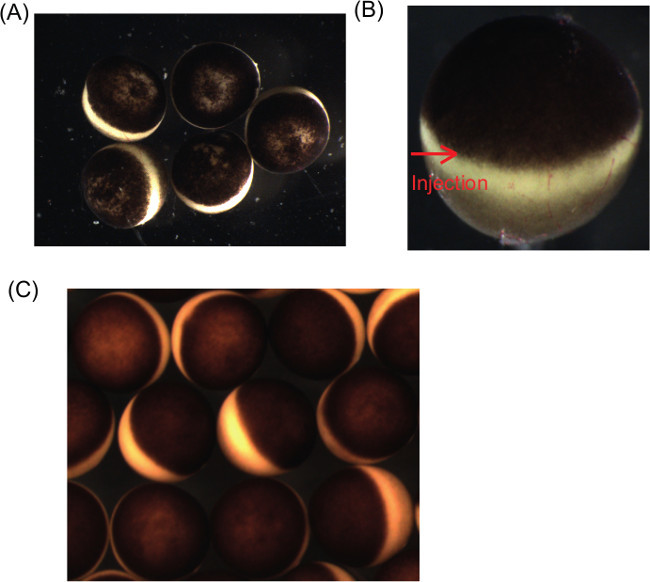
**Figure 1: Typical examples of *****Xenopus laevis***** oocytes of good quality or bad quality for *****in vitro***** maturation experiments. (A)** An example of bad quality oocytes. For example, oocytes showing patchy pigmentation are not used for subsequent experiments. Each *Xenopus laevis* oocyte is approximately 1.2-1.4 mm in diameter.** (B)** A partially defolliculated oocyte. The right half of the oocyte is covered by follicle cells, which can be discerned by the presence of blood vessels. An arrow shows an area free of follicle cells and into which the injection needle is injected. **(C)** An example of good quality oocytes, which are equally sized and show evenly pigmented animal hemispheres with clear contrast between the animal hemisphere and the vegetal hemisphere.


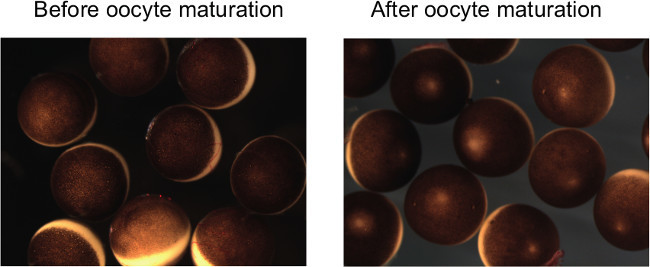
**Figure 2: *****Xenopus laevis***** oocytes before and after maturation. **After oocyte maturation, clear white spots appear at the top of animal hemispheres and follicle cells are peeled off. Each *Xenopus laevis* oocyte is approximately 1 mm in diameter.


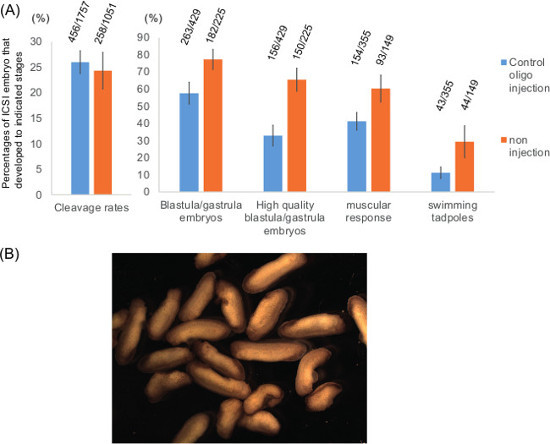
**Figure 3: Development of ICSI embryos, produced from *****in vitro***** matured oocytes. (A)** Development of ICSI embryos to each stage is summarized. Oocytes were injected with control scrambled antisense oligonucleotides (control oligo injection) or without injection (non injection), followed by IVM and ICSI. The corresponding number of embryos at each stage is shown above the bars. Mean ± SEM is shown. N = 4-13 independent experiments/different females.** (B) **Examples of surviving ICSI embryos. These tailbud stage embryos were produced in one experiment, in which approximately 200 oocytes were used as a starting material.

## Discussion

We here detail a new method to deplete maternal factors or to overexpress exogenous factors before fertilization. This system requires two microinjections, but instead skips the surgery of frogs, used in the host transfer method^7,17^. It is optimal to remove the frog surgery step in terms of animal care. Moreover, we do not have to take into account for the quality of host female frogs for the transfer experiments, meaning that we can get rid of one biological factor that affects the success of experiments. Therefore in this system the quality of oocytes obtained from PMSG-primed frogs is a major biological factor that is key for successful experiments. The quality of oocytes can be judged after collection of ovary or after defolliculation. If any abnormality is observed at these stages, it is optimal to collect another ovary from a new frog. Another point when you can check the oocyte quality is after oocyte maturation. If less than 80% of progesterone-treated oocytes show signs of maturation, you may not be able to obtain the enough number of embryos for further analyses. The use of enzymatic defolliculation instead of manual defolliculation is also another advantage of using this system to reduce the labor required for defolliculation. However, it is still possible that manual defolliculation may give a better development than the enzymatic treatment.

As shown in **Figure 3**, almost 10% of *in vitro* matured oocytes can reach the swimming tadpole stage. These data are collected from 13 independent experiments including those reported in ^8^ and new injection experiments. Host transfer method needs 75-150 oocytes in each treatment for obtaining meaningful data since 30-60% of the transferred oocytes can reach the neurula stage^17^. Our method normally starts with 200-300 oocytes in each experimental group since approximately 40-60% of cleaved embryos can reach the muscular response stage. These data suggest that both methods support a reasonable developmental rate. We have so far obtained 10 alive frogs from control mRNA-injected and control antisense oligo-injected oocytes using this technique, indicating that this approach supports development through metamorphosis.

Our oocyte manipulation-ICSI method provides an opportunity to test the role of maternal factors during very early embryonic development, soon after fertilization. In addition, overexpression of chromatin modifying factors before fertilization may make it possible to remodel maternal chromatin before fertilization for understanding maternal chromatin states necessary for development. This strategy may also work well with current gene editing systems such as Transcription activator-like effector nuclease (TALEN)^18,19^ and CRISPR/CAS9^20,21^ since these can be expressed even before fertilization. Therefore, our new approach has a potential to be used for many applications in future.

## Disclosures

The authors have nothing to disclose.
